# The combination of Ki67, histological grade and estrogen receptor status identifies a low-risk group among 1,854 chemo-naïve women with N0/N1 primary breast cancer

**DOI:** 10.1186/2193-1801-2-111

**Published:** 2013-03-14

**Authors:** Carina Strand, Martin Bak, Signe Borgquist, Gunilla Chebil, Anna-Karin Falck, Marie-Louise Fjällskog, Dorthe Grabau, Ingrid Hedenfalk, Karin Jirström, Marie Klintman, Per Malmström, Hans Olsson, Lisa Rydén, Olle Stål, Pär-Ola Bendahl, Mårten Fernö

**Affiliations:** 1 Division of Oncology, Department of Clinical Sciences Lund, Skåne University Hospital, Lund University, Barngatan 2B, SE-221 85 Lund, Sweden; 2 Department of Pathology, Odense University Hospital, Odense, DK-5000 Denmark; 3 Unilabs, Mammography, Bergaliden, SE-252 23 Helsingborg, Sweden; 4 Department of Surgery, Helsingborg Hospital, SE-281 85 Helsingborg, Sweden; 5 Department of Oncology, Radiology and Clinical Immunology, Uppsala University, SE-751 85 Uppsala, Sweden; 6 Department of Pathology, Skåne University Hospital, Lund University, SE-221 85 Lund, Sweden; 7 Division of Pathology, Department of Clinical Sciences Lund, Lund University, SE-221 85 Lund, Sweden; 8 Skåne Department of Oncology, Skåne University Hospital, SE-221 85 Lund, Sweden; 9 Department of Clinical and Experimental Medicine, Faculty of Health Sciences, Department of Clinical Pathology and Clinical Genetic, County Council of Östergötland, Linköping University, Molecular and Immunological Pathology, SE-581 91 Linköping, Sweden; 10 Division of Surgery, Department of Clinical Sciences Lund, Skåne University Hospital, Lund University, SE-221 85 Lund, Sweden; 11 Division of Oncology, Department of Clinical and Experimental Medicine, Faculty of Health Sciences, County Council of Östergötland, Linköping University, SE-581 85 Linköping, Sweden

**Keywords:** Breast cancer, Chemo-naïve, Ki67, Prognostic index, Proliferation

## Abstract

**Background:**

The aim was to confirm a previously defined prognostic index, combining a proliferation marker, histological grade, and estrogen receptor (ER) in different subsets of primary N0/N1 chemo-naïve breast cancer patients.

**Methods/design:**

In the present study, including 1,854 patients, Ki67 was used in the index (KiGE), since it is the generally accepted proliferation marker in clinical routine. The low KiGE-group was defined as histological grade 1 patients and grade 2 patients which were ER-positive and had low Ki67 expression. All other patients made up the high KiGE-group. The KiGE-index separated patients into two groups with different prognosis. In multivariate analysis, KiGE was significantly associated with disease-free survival, when adjusted for age at diagnosis, tumor size and adjuvant endocrine treatment (hazard ratio: 3.5, 95% confidence interval: 2.6–4.7, *P*<0.0001).

**Discussion:**

We have confirmed a prognostic index based on a proliferation marker (Ki67), histological grade, and ER for identification of a low-risk group of patients with N0/N1 primary breast cancer. For this low-risk group constituting 57% of the patients, with a five-year distant disease-free survival of 92%, adjuvant chemotherapy will have limited effect and may be avoided.

## Introduction

To predict clinical outcome and the effect of adjuvant systemic treatment in breast cancer, recommendations such as the St. Gallen breast cancer consensus guidelines (Goldhirsch et al. 2011) and the Adjuvant! Online tool (Ravdin et al. 2001) can be used. The development of array-based technologies and sequencing of the human genome (Perou et al. 2000; Sorlie et al. 2001; Paik et al. 2004; Sotiriou et al. 2006; Ivshina et al. 2006), provide additional information beyond the traditional criteria used to guide treatment decisions. This challenges the currently used factors, such as lymph node involvement, tumor size, age, histological grade, human epidermal growth factor receptor 2 (HER2), Ki67, and estrogen (ER) and progesterone receptor (PgR) status (Goldhirsch et al. 2011; Aebi et al. 2011; Harris et al. 2007). At the St. Gallen Consensus Meeting in 2011, Onco*type*DX^®^ was considered useful for predicting responsiveness to chemotherapy in an endocrine-responsive cohort, whereas other tests were considered not yet fully validated. Two of the most well-known gene-based assays, MammaPrint^®^ and Onco*type*DX^®^ have, however, also been questioned with regard to their added prognostic value (Edén et al. 2004; Cuzick et al. 2011). The ongoing clinical trials, MINDACT and TAILORx, will hopefully provide more conclusive data (Rutgers et al. 2011; Zujewski & Kamin 2008).

High proliferation is a key feature in breast carcinogenesis and markers of proliferation have been shown to be associated to prognosis and to effect of adjuvant and palliative chemotherapy, to neoadjuvant endocrine therapy, and to prognosis after adjuvant endocrine treatment (Harris et al. 2007; Colozza et al. 2005; Beresford et al. 2006; De Azambuja et al. 2007; Goldhirsch et al. 2009; Urruticoechea et al. 2005; Hietanen et al. 1995; Amadori et al. 2008; Jones et al. 2009; Ellis et al. 2008; Viale et al. 2008). Ki67 is the marker of proliferation most widely used (Goldhirsch et al. 2011; De Azambuja et al. 2007; Urruticoechea et al. 2005; Dowsett et al. 2011; Luporsi et al. 2012), but the role of other markers, such as cyclin A (Bukholm et al. 2001; Michalides et al. 2002; Kuhling et al. 2003; Baldini et al. 2006; Ahlin et al. 2009; Strand et al. 2012) and phosphohistone H3 (Skaland et al. 2007), remains under debate. Furthermore, global gene expression analyses have shown that proliferation-associated genes seem to be among the most important for dividing patients into groups with different prognosis, especially in ER-positive and histological grade 2 breast cancers (Sotiriou et al. 2006; Ivshina et al. 2006; Teschendorff et al. 2007; Desmedt et al. 2008). In line with this, studies from our group, as well as others, have shown that single markers of proliferation (Ki67 and cyclin A) were of prognostic importance in ER-positive breast cancer and in the histological grade 2 (Strand et al. 2012; Klintman et al. 2010) and grade 1 (Aleskandarany et al. 2011) subgroups. We have previously evaluated the importance of a prognostic index based on the combination of cyclin A, histological grade, and ER (CAGE), in node-negative premenopausal breast cancer patients (Strand et al. 2012). The CAGE-index combined with HER2-status classified 53% of the women as low-risk patients with a five-year distant disease-free survival (DDFS) of 95%. For this low-risk group, adjuvant cytotoxic treatment will have limited efficacy and may be avoided. In the present study, we present data for Ki67. Importantly, avoiding unnecessary use of chemotherapy for low-risk patients with N0/N1 primary breast cancer is also the primary aim of the MINDACT and TAILORx trials (Rutgers et al. 2011; Zujewski & Kamin 2008).

The aim of this study was to confirm a prognostic index based on the combination of proliferation (Ki67), histological grade, and ER (KiGE) in different subsets of chemo-naïve patients with N0/N1 primary breast cancer with special focus on five-year DDFS.

## Materials and methods

### Patients

We included 1,854 women with primary breast cancer of which 1,522 originated from two randomized clinical studies (Patient materials I–II) and three cohorts (Patient materials III–V). The remaining 332 patients came from a case–control study (Patient material VI). Patients were excluded due to adjuvant chemotherapy and/or missing information on adjuvant therapy, Ki67, histological grade, or ER. Furthermore, for patients with more than three positive lymph nodes, adjuvant chemotherapy is recommended (as stated in the St. Gallen guidelines (Goldhirsch et al. 2011), hence these patients were also excluded. The endpoint for the 1,522 patients was defined as distant recurrence for 86% of the patients (Patient materials I–II and IV–V) and as any recurrence for the remaining 14% (Patient material III). Time to this endpoint will hereafter be referred to as event-free survival. Median follow-up for patients alive and event-free at last follow-up was 7.2 years (range: 1.1–17 years). Only the first five years of follow-up were used in the analyses.

#### Patient material I

SBII:2-pre (*N*=221, 68 distant recurrences). Premenopausal women with stage II breast cancer were enrolled, between 1986 and 1991, in a randomized trial with the aim to compare the effect of two years of tamoxifen (TAM) treatment versus no adjuvant systemic treatment. The original trial included 564 patients enrolled in the South and South-East Swedish Health Care Regions (Rydén et al. 2005).

#### Patient material II

SBII:2-post (*N*=166, 22 distant recurrences). Postmenopausal women with stage II breast cancer were enrolled, between 1983 and 1991, in a randomized trial launched by the Swedish Breast Cancer group of two versus five years of adjuvant TAM (Swedish Breast Cancer Cooperative Group 1996). The original trial included 1,107 patients from the South Swedish Health Care Region. Paraffin embedded tumor material has previously been collected from a subgroup of patients treated with TAM for two years, for comparison of a cytosol method and immunohistochemistry for analyses of ER and PgR (Chebil et al. 2003). In the present study, the paraffin embedded material was used for analyses of Ki67 and HER2, and for the re-evaluation of histological grade.

#### Patient material III

The Malmö cohort (*N*=217, 32 recurrences). The original cohort enrolled a consecutive series of 498 patients diagnosed with primary breast cancer at the Department of Pathology, Malmö University Hospital between 1988 and 1992. The purpose was to construct tissue microarrays for biomarker evaluation (Borgquist et al. 2008).

#### Patient material IV

The Bone marrow metastases cohort (*N*=379, 27 distant recurrences). The original study included 569 consecutive patients diagnosed with primary breast cancer in the South Swedish Health Care Region and included patients diagnosed between 1999 and 2003. The purpose was to study the prognostic value of the presence of cytokeratin positive cells in bone marrow aspirates from the sternum (Falck et al. 2012).

#### Patient material V

The Odense cohort (*N*=539, 86 distant recurrences). The original study enrolled a consecutive series of 841 patients with primary breast cancer referred to Odense University Hospital, Denmark. Patients were enrolled between 1980 and 1990 (Hansen et al. 2000). The purpose was to collect a population-based cohort for evaluation of prognostic factors.

All patients from these collections (Patient materials I–V) were pooled in a database from which the following subsets were extracted: *Set 1*: node-negative (N0), no adjuvant therapy, ≤50 years at diagnosis (*N*=169, 20 events), *Set 2*: N0, no adjuvant therapy, >50 years at diagnosis (*N*=488, 55 events), *Set 3*: node-positive (N1), no adjuvant therapy (*N*=167, 39 events), *Set 4*: N0, adjuvant endocrine therapy (*N*=291, 39 events), and *Set 5*: N1, adjuvant endocrine therapy (*N*=407, 82 events) (Table 1). The reason for the subdivision with regard to age between Sets 1 and 2 was to confirm the index from our original study (Strand et al. 2012) in a corresponding subgroup with regard to menopausal status, adjuvant therapy and lymph node status.

**Table 1:** **Patient and tumor characteristics**

Factor		Set 1	Set 2	Set 3	Set 4	Set 5	Total
***No of patients***		169	488	167	291	407	1,522
***Events***		20	55	39	39	82	235
***Patient material I***^**a**^		30	9	66	39	77	221
***Patient material II***^**a**^		0	0	0	67	99	166
***Patient material III***^**a**^		28	100	5	27	57	217
***Patient material IV***^**a**^		16	109	9	145	100	379
***Patient material V***^**a**^		95	270	87	13	74	539
***Age*** median, years		45	65	53	61	61	60
***Age*** range, years		28-50	50-90	27-93	30-88	33-89	27-93
***Tumor size***							
	≤ 20 mm	113 (67)^b^	364 (75)	68 (41)	115 (40)	208 (51)	868 (57)
	>20 mm	56 (33)	124 (25)	99 (59)	176 (60)	199 (49)	654 (43)
***Lymph nodes***							
	Negative	169	488	0	291	0	948 (62)
	1-3 positive	0	0	167	0	407	574 (38)
***ER status***							
	Positive	131 (78)	399 (82)	122 (73)	235 (81)	329 (81)	1,216 (80)
	Negative	38 (22)	89 (18)	45 (27)	56 (19)	78 (19)	306 (20)
***PgR status***							
	Positive	46 (71)	121 (61)	56 (70)	168 (63)	200 (63)	591 (64)
	Negative	19 (29)	76 (39)	24 (30)	100 (37)	118 (37)	337 (36)
	Missing	104	291	87	23	89	594
***Histological grade***							
	1	41 (24)	136 (28)	19 (11)	32 (11)	67 (17)	295 (20)
	2	82 (49)	250 (51)	97 (58)	166 (57)	233 (57)	828 (54)
	3	46 (27)	102 (21)	51 (31)	93 (32)	107 (26)	399 (26)
***Ki67***							
	Low	109 (64)	343 (70)	111 (66)	210 (72)	315 (77)	1,088 (71)
	High^c^	60 (36)	145 (30)	56 (34)	81 (28)	92 (23)	434 (29)
***HER2 status***							
	Negative	54 (92)	142 (87)	62 (84)	216 (86)	249 (88)	723 (87)
	Positive^d^	5 (8)	21 (13)	12 (16)	36 (14)	33 (12)	107 (13)
	Missing	110	325	93	39	125	692
***Adjuvant endocrine treatment***							
	Yes	0	0	0	291	407	698 (46)
	No	169	488	167	0	0	824 (54)

Patient materials I-V were pooled in a database from which the following subsets were extracted: ***Set 1***: node-negative (N0), no adjuvant therapy, ≤50 years at diagnosis, ***Set 2***: N0, no adjuvant therapy, >50 years at diagnosis, ***Set 3***: node-positive (N1), no adjuvant therapy, ***Set 4***: N0, adjuvant endocrine therapy, and ***Set 5***: N1, adjuvant endocrine therapy.

*ER* = estrogen receptor, *PgR* = progesterone receptor, *HER2* = human epidermal growth factor 2.

^a^Patient material I (Rydén et al. 2005), Patient material II (Swedish Breast Cancer Cooperative Group 1996; Chebil et al. 2003), Patient material III (Borgquist et al. 2008), Patient material IV (Falck et al. 2012), and Patient material V (Hansen et al. 2000).

^b^Numbers in parentheses are percentages.

^c^High Ki67 was previously defined as cases above the seventh decile in the empirical Ki67 distribution (which corresponds to 20% positive cells) (Klintman et al. 2010) and 20% was used for Patient materials I-IV. Cases above the median were considered Ki67 high in Patient material V.

^d^HER2: positive if HER2-IHC 3+ or HER2-IHC 2+ and FISH amplified.

#### Patient material VI

The Uppsala study (*N*=166 cases and 166 controls, Table 2). The original study included 900 patients diagnosed with primary breast cancer in the Uppsala-Örebro region from 1993–2004. Exclusion criteria were tumor size >50 mm, lymph node metastases or adjuvant chemotherapy. Within this cohort, cases were defined as women who died from breast cancer. Eligible as controls were patients alive at the time of the corresponding case’s death (Ahlin et al. 2009).

**Table 2:** **Patient and tumor characteristics for the case–control study (Patient material VI** (Ahlin et al. 2009**)**

Factor		Patient material VI	
		Case	Control
***No of patients***		166	166
***Age*** median, years		69	61
***Age*** range, years		34-88	32-89
***Tumor size***			
	≤ 20 mm	102 (61)^a^	131 (79)
	>20 mm	64 (39)	35 (21)
***Lymph nodes***			
	Negative	166	166
***ER status***			
	Positive	94 (57)	131 (79)
	Negative	72 (43)	35 (21)
***PgR status***			
	Positive	69 (42)	113 (68)
	Negative	96 (58)	53 (32)
	Missing	1	0
***Histological grade***			
	1	14 (8)	39 (23)
	2	85 (51)	94 (57)
	3	67 (41)	33 (20)
***Ki67***			
	Low	87 (52)	112 (67)
	High^b^	79 (48)	54 (33)
***HER2 status***			
	Negative	145 (91)	143 (92)
	Positive^c^	14 (9)	13 (8)
	Missing	7	10
***Adjuvant endocrine treatment***			
	Yes	48 (29)	40 (24)
	No	118 (71)	126 (76)

*ER* = estrogen receptor, *PgR* = progesterone receptor, *HER2* = human epidermal growth factor 2.

^a^Numbers in parentheses are percentages.

^b^High Ki67 was defined as cases with more than 20% positive cells.

^c^HER2: positive if HER2-IHC 3+ or HER2-IHC 2+ and FISH amplified.

### Biomarker analysis and definition of KiGE

ER, PgR, Ki67, HER2, and histological grade were analyzed and evaluated as described elsewhere (Ahlin et al. 2009; Rydén et al. 2005; Chebil et al. 2003; Borgquist et al. 2008; Falck et al. 2012). If previously defined cut-points were available, they were used in the present study. Hence, cases above the median were considered Ki67 high in Patient material V. Furthermore, Ahlin et al. defined high Ki67 as cases above the seventh decile of the empirical Ki67 distribution (which corresponded to 20% positive cells) (Klintman et al. 2010) and therefore 20% was used for Patient materials I–IV and VI. For Patient materials I–IV and VI, Ki67 was evaluated on TMAs and for Patient material V on whole tissue sections. In order to confirm the combination of proliferation, histological grade, and ER, the previously applied index (CAGE) was used (Strand et al. 2012), but cyclin A was replaced by Ki67, thereby creating KiGE. The low KiGE-group was defined as histological grade 1 patients and grade 2 patients which were ER-positive and had low Ki67 expression. High KiGE consisted of all other patients.

### Statistical methods

The Kaplan-Meier method was used to estimate event-free survival and the Cox proportional hazards model, stratified by patient material, was used for estimation of hazard ratios (HR:s). Proportional hazards assumptions were checked with Schoenfeld’s test (Schoenfeld 1983). To avoid severe problems with non-proportional hazards, the follow-up was restricted to the first five years after diagnosis.

In Patient material VI, conditional logistic regression analysis was used to estimate odds ratios (OR:s) and confidence intervals (CI:s), using the proportional hazards regression procedure in statistical analysis software (SAS).

Forest plots were used to visualize HR:s and 95% CI:s for the different subsets and the overall measure of effect which was estimated using a DerSimonian-Laird random-effects model.

All tests were two-sided. For evaluation of the primary aim, the effect of the KiGE-index, *P*-values <0.01 were considered significant. The statistical analysis software Stata 12.1, 2012 (StataCorp, College Station, TX) and SAS (SAS Institute, Inc.) were used for statistical calculations. Whenever applicable, the REMARK recommendations for reporting of tumor marker studies were followed (McShane et al. 2005). The study was approved by the ethics committee at Lund University (LU 240-01).

## Results

### KiGE evaluation

#### Univariate analyses

**Figure 1:**
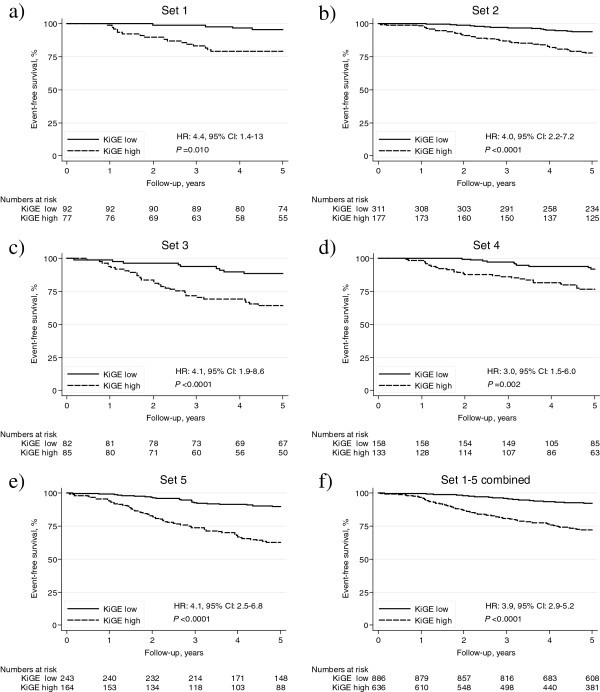
**Kaplan**-**Meier survival estimates of event**-**free survival**, **and hazard ratios(HR)with corresponding 95%confidence intervals(CI)for the different subsets(1a-e),stratified by patient material**, **and for all the patients(1f).** From Patient materials I-V the following subsets were extracted: ***Set 1***: node-negative (N0), no adjuvant therapy, ≤50 years at diagnosis (1**a**), ***Set 2***: N0, no adjuvant therapy, >50 years at diagnosis (1**b**), ***Set 3***: node-positive (N1), no adjuvant therapy (1**c**), ***Set 4***: N0, adjuvant endocrine therapy (1**d**), and ***Set 5***: N1, adjuvant endocrine therapy (1**e**). Event-free survival corresponds to distant disease-free survival for Patient materials I-II and IV-V, and to recurrence-free survival for Patient material III.

**Figure 2:**
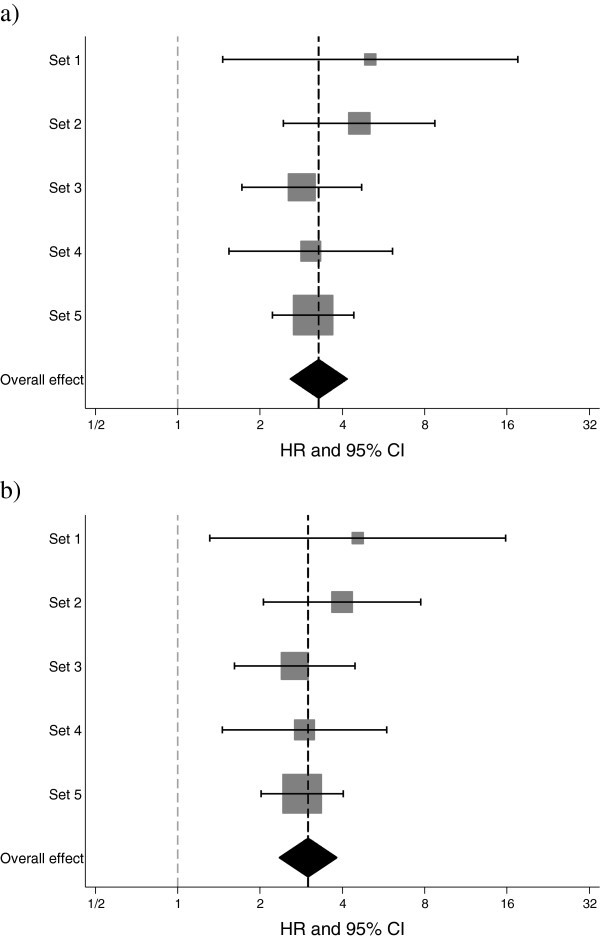
**Forest plots for the different subsets, showing hazard ratios (HR:s) with corresponding 95% confidence intervals (CI:s) for KiGE in univariate analysis (a) and in multivariate analysis (b), adjusted for age at diagnosis and tumor size.** The diamonds and the vertical dashed lines represent the overall measures of effect. The areas of the grey squares are proportional to each subset’s weight in the meta-analysis.

The HR:s for high KiGE versus low KiGE, when each set (Set 1–5) was analyzed separately, varied between 3.0 and 4.4, being statistically significant for all sets (Figures 1a–e and 2a). When including all patients in Set 1–5 (*N*=1,522, 235 events), a statistically significant association between the combination variable KiGE and event-free survival was found (HR: 3.9, 95% CI: 2.9–5.2, *P*<0.0001; Figure 1f).

In the case–control study (Patient material VI), there was a statistically significant association between the combination variable KiGE and breast cancer death (OR: 2.7, 95% CI: 1.7–4.3, *P*<0.0001).

#### Multivariate analyses

The HR:s for KiGE for each of the five subsets, when adjusted for age at diagnosis and tumor size, were similar compared to those without adjustment, as illustrated by the forest plot (Figure 2b). When all subsets (Set 1–5) were included, KiGE was significantly associated with event-free survival, after adjustment for age at diagnosis, tumor size, and adjuvant endocrine treatment (HR: 3.5, 95% CI: 2.6–4.7, *P*<0.0001). Including HER2 in the multivariate analysis (*N*=830, 133 events), KiGE remained significantly associated with event-free survival (HR: 4.0, 95% CI: 2.7–6.0, *P*<0.0001).

In the case–control study, KiGE was significantly associated with breast cancer death, after adjustment for age at diagnosis, tumor size, and adjuvant endocrine treatment (OR: 3.2, 95% CI: 1.8–5.5, *P*<0.0001).

### Subset analyses of Ki67 in patients not treated with adjuvant tamoxifen (Set 1–3)

#### *Ki67 in ER*-*positive* versus *ER*-*negative cases*

In order to confirm results from previous investigations (Teschendorff et al. 2007; Desmedt et al. 2008; Klintman et al. 2010) showing that the prognostic importance of Ki67 is dependent on ER-status, subgroup analyses were performed, stratified by ER-status. In univariate analyses, Ki67 was a significant prognostic factor in the ER-positive subgroup (HR: 3.3, 95% CI: 2.0–5.5, *P*<0.0001; *N*=652, 76 events), but not in the ER-negative subgroup (HR: 1.0, 95% CI: 0.49–2.1, *P*=0.96; *N*=172, 38 events). The difference in prognostic importance of Ki67 between ER-positive and ER-negative cases was further analyzed in a Cox model allowing for interaction between the two factors. The interaction effect, corresponding to the ratio of the HR:s for Ki67 in the ER-positive and ER-negative subgroups, was 2.7 (95% CI: 1.2–6.1, *P*=0.02).

#### Ki67 in histological grade subgroups

Previous studies have demonstrated that the prognostic importance of Ki67 is mainly attributed to the histological grade 2 subgroup (Klintman et al. 2010; Aleskandarany et al. 2011). Similar trends were seen in the present study. In the histological grade 2 tumors (*N*=429, 51 events), the HR for high versus low Ki67 was 1.8 (95% CI: 1.0–3.4, *P*=0.05). The five-year event-free survival figures were 82% (95% CI: 73–88%) for high Ki67 and 90% (95% CI: 86–93%) for low Ki67, respectively. There were too few events (*N*=196, 5 events) to draw any conclusions on the impact of Ki67 in the histological grade 1 subgroup. The five-year event-free survival in the histological grade 1 subgroup was 97% (95% CI: 93–99%), independent of Ki67. Ki67 was not a significant prognostic factor in the histological grade 3 subgroup (HR: 1.0, 95% CI: 0.56–1.9, *P*=0.91; *N*=199, 58 events), with five-year DDFS of 73% (95% CI: 64–79%) and 65% (95% CI: 52–76%) for high and low Ki67 groups, respectively.

### Five-year DDFS for high and low KiGE (Patient materials I–II and IV–V)

**Figure 3:**
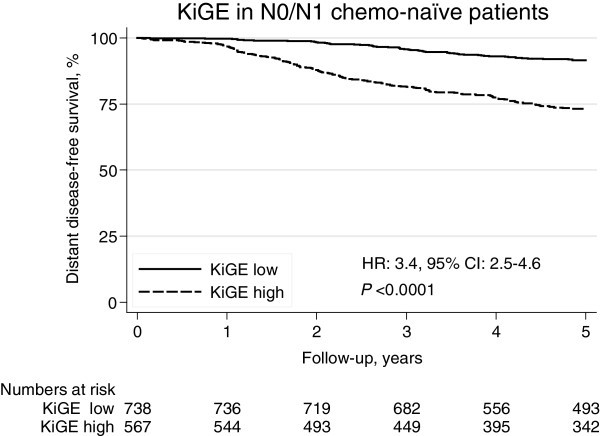
**Five-year DDFS by the KiGE-index in N0/N1 chemo-naïve breast cancer patients.**

Use of the KiGE-index in the N0/N1-subgroup with information on distant recurrences (*N*=1,305, 203 distant recurrences) identified a low-risk group constituting 57% of the patients, with a five-year DDFS of 92% (95% CI: 89–93%). The DDFS for the remaining 43% of the patients was 73% (95% CI: 69–77%). The association between the KiGE-index and DDFS was statistically significant (HR: 3.4, 95% CI: 2.5–4.6, *P*<0.0001; Figure 3).

In the N0-subgroup (*N*=793, 97 distant recurrences), the KiGE-index identified a low-risk group constituting 57% of the patients with a five-year DDFS of 93% (95% CI: 90–95%; HR: 3.2, 95% CI: 2.0–4.9, *P*<0.0001). Equally, in the N1-subgroup (*N*=512, 106 distant recurrences), the KiGE-index identified a low-risk group of similar size (56%) with a five-year DDFS of 89% (95% CI: 85–92%; HR: 3.7, 95% CI: 2.4–5.7, *P*<0.0001).

## Discussion

This confirmation study for N0/N1 chemo-naïve breast cancer patients, confirms the prognostic value of a previously defined index combining proliferation (previously cyclin A, in the present study Ki67), histological grade, and ER. Importantly, the KiGE-index separated chemo-naïve patients into groups with different risk, independent of menopausal status, lymph node status, and whether endocrine adjuvant treatment was given or not. The robustness of the index is strengthened by the fact that the evaluation of Ki67, histological grade, and ER was performed in different studies by different persons using different cut-points, that studies from three Swedish health care regions and one Danish region were included, and by the fact that different study designs were used (randomized, cohort and case-control studies). Furthermore, when analyzing Patient materials I–V separately (not the five sets), KiGE remained a significant prognostic factor (HR:s varied between 2.1 and 9.0) in all but one patient material (Patient material II, *P*=0.09; data not shown). We were also able to confirm the previous finding that the prognostic value of Ki67 is limited to ER-positive breast cancer and is most pronounced for the histological grade 2 subgroup. The latter findings are furthermore in line with gene expression analyses (Sotiriou et al. 2006; Ivshina et al. 2006; Teschendorff et al. 2007; Desmedt et al. 2008). However, a recent publication (Munzone et al. 2012) showed that within the group of patients with node-negative triple-negative breast cancer, Ki67 was associated with different prognosis when using a higher cut-point (35%). The KiGE-index is similar to the index proposed at the St. Gallen consensus meeting in 2011 (Goldhirsch et al. 2011), with Ki67 separating clinicopathologically classified ‘Luminal’ ER-positive breast cancer into ‘Luminal A’ and ‘Luminal B’ subgroups with different prognoses and thereby influence on the choice of therapy. Chemotherapy, with or without anti-HER2 therapy, is suggested for the ‘Luminal B’ subgroup, but not for the ‘Luminal A’ subgroup (Goldhirsch et al. 2011). According to the St. Gallen guidelines, not taking histological grade into consideration, patients with ER-positive low proliferating (and HER2 normal) tumors will be classified as having ‘Luminal A’ breast cancer. According to the KiGE-index, with the inclusion of histological grade, some of these patients will instead be considered as having worse prognosis. In this large patient material, patients with ER-positive, low Ki67, and histological 3 breast cancer (*N*=42) have a poor prognosis, with a five-year DDFS of 64% (95% CI: 47–76%). This subgroup constituted 6% (42/652) of the ER-positive (chemo-naïve) patients in our study (Patient materials I–V). In a recent publication by the International Ki67 in Breast Cancer Working Group (Dowsett et al. 2011), certain important drawbacks for Ki67 analyses were highlighted, including number of cancer cells being scored and cut-point used. Furthermore, the distribution of Ki67 values makes it difficult to define a cut-point. The inherent drawbacks with the evaluation of Ki67 may at least partly be overcome by also considering histological grade, as suggested in the present study. Our study was, probably due to low power, unable to demonstrate any prognostic importance of Ki67 in histological grade 1 breast cancer. Aleskandarany et al. however, demonstrated that patients with high Ki67 had a significantly worse prognosis than those with low Ki67, in a large study, including 494 histological grade 1 breast cancers (Aleskandarany et al. 2011). Therefore, we do not exclude the possibility that Ki67 has prognostic value in the histological grade 1 subgroup.

Identifying a subgroup of patients not in need of adjuvant chemotherapy, was found to be the top priority on a list of the most urgent research areas in breast cancer in a recent web-consultant study (Dowsett et al. 2007). The Early Breast Cancer Trialists´ Collaborative Group (Peto et al. 2012) demonstrated that the effect of chemotherapy was independent of age, node status, tumor size, differentiation, ER-status, and tamoxifen use. However, information on quantitative immunohistochemistry of proliferation was not included. Ki67 combined with ER-status and histological grade may be helpful in this respect. Breast cancer patients with histological grade 1 tumors or patients with ER-positive and histological grade 2 tumors with low Ki67 expression constitute 57% of the patients with N0/N1 cancers in this study, with a five-year DDFS of 92%. Adjuvant chemotherapy would have limited added value for this group. The identification of a low-risk group not in need of adjuvant chemotherapy is also the primary aim of two ongoing clinical trials (MINDACT and TAILORx) (Rutgers et al. 2011; Zujewski & Kamin 2008) evaluating the gene profiles MammaPrint^®^ and Onco*type*DX^®^. The MINDACT trial (Rutgers et al. 2011) is a prospective, randomized trial using the 70-gene signature (MammaPrint^®^) together with the common clinical-pathological criteria for selecting patients for adjuvant chemotherapy. It is being tested in patients with N0/N1 breast cancer. MINDACT has a null hypothesis of a five-year distant metastasis-free survival of 92%, which will be tested for the group of patients who have a low-risk gene prognosis signature and high clinical-pathological criteria, and who were randomized to use the 70-gene signature and thus receive no chemotherapy. In a pilot phase based on the first 800 patients from this trial, the low-risk group constituted 65% (Rutgers et al. 2011). This figure is in line with 57% being the percentage of low-risk patients in our study, who furthermore had the same five-year DDFS as the null hypothesis in the MINDACT trial (92%). Risk group stratification may be further modified by also considering other established prognostic factors, e.g. lymph node status, tumor size, age, and HER2-status. The incidence of recurrences in the low-risk group could thereby be further decreased, but with the consequence of a smaller low-risk group.

In conclusion, we have confirmed a prognostic index based on proliferation (Ki67), histological grade, and ER for identifying a low-risk group of patients with N0/N1 breast cancer. For this large low-risk group, with a five-year DDFS of 92%, adjuvant chemotherapy will have limited effect and may thus be avoided.
